# Peptide-Modified Surfaces for Enzyme Immobilization

**DOI:** 10.1371/journal.pone.0018692

**Published:** 2011-04-08

**Authors:** Jinglin Fu, Jeremy Reinhold, Neal W. Woodbury

**Affiliations:** 1 Center for Single Molecule Biophysics, Arizona State University, Tempe, Arizona, United States of America; 2 Center for Innovations in Medicine, the Biodesign Institute, Arizona State University, Tempe, Arizona, United States of America; 3 Department of Chemistry and Biochemistry, Arizona State University, Tempe, Arizona, United States of America; Deutsches Krebsforschungszentrum, Germany

## Abstract

**Background:**

Chemistry and particularly enzymology at surfaces is a topic of rapidly growing interest, both in terms of its role in biological systems and its application in biocatalysis. Existing protein immobilization approaches, including noncovalent or covalent attachments to solid supports, have difficulties in controlling protein orientation, reducing nonspecific absorption and preventing protein denaturation. New strategies for enzyme immobilization are needed that allow the precise control over orientation and position and thereby provide optimized activity.

**Methodology/Principal Findings:**

A method is presented for utilizing peptide ligands to immobilize enzymes on surfaces with improved enzyme activity and stability. The appropriate peptide ligands have been rapidly selected from high-density arrays and when desirable, the peptide sequences were further optimized by single-point variant screening to enhance both the affinity and activity of the bound enzyme. For proof of concept, the peptides that bound to β-galactosidase and optimized its activity were covalently attached to surfaces for the purpose of capturing target enzymes. Compared to conventional methods, enzymes immobilized on peptide-modified surfaces exhibited higher specific activity and stability, as well as controlled protein orientation.

**Conclusions/Significance:**

A simple method for immobilizing enzymes through specific interactions with peptides anchored on surfaces has been developed. This approach will be applicable to the immobilization of a wide variety of enzymes on surfaces with optimized orientation, location and performance, and provides a potential mechanism for the patterned self-assembly of multiple enzymes on surfaces.

## Introduction

Surface-immobilized enzymes play an important role in many biocatalytic processes and industrial applications [1], [2]. The activity, stability and selectivity of enzymes can be improved if they are immobilized properly on surfaces [1], [3]. Many conventional protein immobilization methods [1], which rely on nonspecific absorption of proteins to solid supports or chemical coupling of reactive groups within proteins, have inherent difficulties, such as protein denaturation, poor stability due to nonspecific absorption [4], [5], variations in the spatial distance between enzymes and the enzyme-to-surface distance [6], and the inability to control protein orientation [1], [5]. New strategies for enzyme immobilization are needed which allow the precise control over orientation and position and thereby provide optimized activity [7]. Peptides represent a promising class of potential protein-anchoring/modulating molecules due to their large chemical diversity [8] and the existence of well-established methods for library synthesis [9]. There is a growing realization that, by using peptides as building blocks, it is possible to create synthetic structures with affinities and specificities comparable to natural antibodies [10], [11]. Peptide or small molecule ligands that bind to a unique region of a protein can be used for orienting the protein and modulating its activity through specific ligand-protein interactions on a solid support [11]–[13]. In this work, we present a method for creating peptide-modified surfaces that immobilize a target enzyme with optimized orientation and activity.

## Results

**Figure 1:**
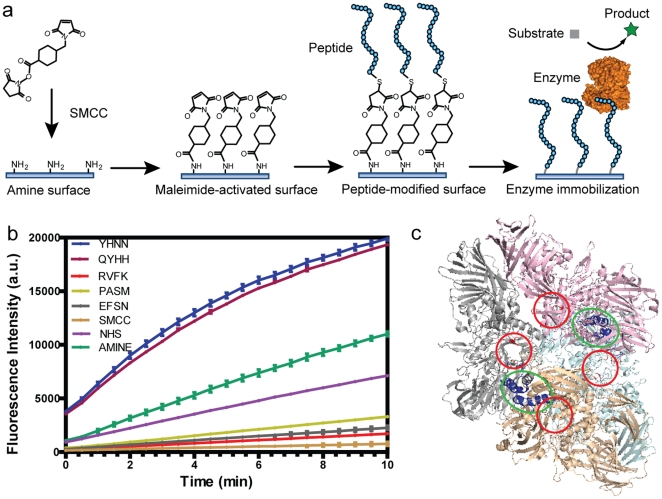
Enzyme immobilization on peptide-modified surfaces. (a) The overall process for conjugating peptides to aminated microwells through specific reactions between C-terminal cysteines and maleimide-activated surfaces. (b) Activity of β-Gal immobilized on different surfaces. 25 nM β-Gal is first incubated with modified microwells for one hour and then enzyme activity is measured at 25°C as a function of time using 100 µM Resorufin β-D-galactopyranoside as the substrate. YHNN, QYHH, RVFK, PASM and EFSN represent β-Gal bound to various peptide-modified surfaces (see text). SMCC and NHS represent enzyme covalently bound via thiol and amine conjugation, respectively. AMINE represents enzyme bound noncovalently to an aminated surface. (c) Proteolytic mapping of peptide binding to tetrameric β-Gal with binding regions circled (Green). Each subunit is labeled with a unique color showing the symmetry of the β-Gal structure. The binding regions (amino acids 419–447) are highlighted in blue. The substrate-binding sites of β-Gal are circled with right color (Glu461, Met502, Tyr503 and Glu537) according to reference 16.

Previously, we described an approach for screening high-density peptide arrays to identify specific peptide sequences that anchor enzymes to surfaces and modulate their activity [13]. To demonstrate the utility of this approach more generally for optimized enzyme immobilization, two 20-mer peptides, YHNNPGFRVMQQNKLHHGSC (referred to as YHNN) and QYHHFMNLKRQGRAQAYGSC (referred to as QYHH) were selected from a microarray of 10,000 peptides based on their ability to bind β-galactosidase (β-gal) and optimize its surface-immobilized activity (Table S1 in File S1). These peptides were then synthesized and covalently conjugated to aminated microwells, modifying the surface and mediating the binding of β-gal through specific peptide-enzyme interactions (Figure 1a). As controls, two inhibitory peptides, RVFKRYKRWLHVSRYYFGSC (RVFK) and PASMFSYFKKQGYYYKLGSC (PASM), and one weak-binding peptide, EFSNPTAQVFPDFWMSDGSC (EFSN), were also used to modify aminated microwells (Table S1 in File S1). β-Gal immobilized on YHNN- and QYHH-surfaces exhibited much higher activity than β-Gal immobilized on control peptide-modified surfaces (Figure 1b). The relative specific activities of β-Gal immobilized on peptide-modified microwells were shown in Table 1, which were calculated for each surface by dividing the total bound enzyme activity by the total binding intensity. Conventional surface immobilization approaches were also tested including SMCC-activated (SMCC 6) and NHS-activated (NHS 7) covalent attachment, as well as noncovalent amine-surface attachment (Amine 8). In Table 1, YHNN- and QYHH– modified surfaces resulted in a specific activity of bound enzyme that was ∼2-fold greater than amine noncovalent binding and nearly 3-fold greater than NHS attachment. In addition, the YHNN- and QYHH–modified surfaces have the advantage of specifically associating with β-Gal in a protein mixture. This was shown by binding β-Gal in a solution containing 3% Bovine serum albumin (BSA). YHNN- and QYHH– modified surfaces showed 15-fold more bound enzyme activity than the amine surface and 20-fold more than the NHS surface (Figure S1 in File S1).

**Table 1:** Normalized activity and affinity of β-Gal immobilized on surface-modified microwells^a^.

Surface for protein immobilization	Binding affinity (Norm.)	Activity (Norm.)	Specific activity (Norm.)
1 YHNNPGFRVMQQNKLHHGSC	0.9±0.03	2.1±0.1	2.2±0.1
2 QYHHFMNLKRQGRAQAYGSC	0.9±0.02	2.3±0.1	2.4±0.2
3 RVFKRYKRWLHVSRYYFGSC	0.9±0.05	0.1±0.01	0.1±0.01
4 PASMFSYFKKQGYYYKLGSC	0.5±0.1	0.3±0.06	0.5±0.1
5 EFSNPTAQVFPDFWMSDGSC	0.1±0.01	0.05±0.01	0.4±0.1
6 SMCC	0.2±0.02	0.03±0.01	0.1±0.1
7 NHS	0.8±0.1	0.6±0.1	0.8±0.1
8 Amine	1.0±0.1	1.0±0.1	1.0±0.1

a Types of surfaces: 1 and 2 are selected peptide-modified surfaces; 3 and 4 are control surfaces modified by inhibitory peptides; 5 is a control surface conjugated with a weak-binding peptide; 6–8 are the conventional surfaces used for covalent or noncovalent enzyme immobilization, defined as in Figure 1, legend. All of the data is normalized to that of the amine surface, 8.

In addition, YHNN- and QYHH-modified surfaces were also found to improve the thermal and pH stability of immobilized β-Gal. The thermal stability of bound β-Gal was ∼16-fold greater on the peptide-modified surfaces than free enzyme in solution after incubating at 55°C for one hour (Figure S2 in File S1) and more than 2-fold better than enzyme immobilized to either the NHS or amine surfaces. Immobilization of β-Gal on YHNN- and QYHH-modified surfaces also shifted the pH optimum from pH 8 in free solution to 7 on the surface. Long-term enzyme stability to storage on surfaces was greatly improved on peptide-modified surfaces, particularly when peptide modification was combined with the use of a hydrogel (5% polyvinyl alcohol) coating. β-Gal immobilized in this way and stored dry for one week at room temperature retained ∼35% of its original activity. In contrast, enzyme similarly immobilized and stored on amine surfaces retained less than 5% activity and NHS surfaces retained ∼14% (Figure S3 in File S1). If one considers both the increased binding capacity of the peptide-modified surfaces and their increased stability to storage, there was 20-fold more enzyme activity per surface area after storage on the peptide-modified surfaces than either the amine surfaces or the NHS surfaces, a significant factor in the commercial immobilization and storage of enzymes.

The apparent *K_d_* values of the YHNN- and QYHH-modified surfaces were ∼5 nM and ∼4 nM for β-Gal, respectively (Figure S4 in File S1). The apparent *k_cat_* and *K_m_* constants for immobilized β-Gal were measured on peptide-modified iodoacetyl resin, which has a large binding capacity and allows for the quantification of the absolute amount of bound enzyme (Figure S5 in File S1). *k_cat_* values were ∼46 s^−1^ for the YHNN- surface and ∼53 s^−1^ for the QYHH- surface, similar to the *k_cat_* of ∼58 s^−1^ under the same conditions for the free enzyme. The apparent *K_m_* values of β-Gal bound to the YHNN- and QYHH-modified surfaces were ∼240 µM and 250 µM, respectively, compared ∼130 µM for the free enzyme. The apparent increase in *K_m_* for the surface-bound enzyme may be due to slow diffusion of substrate molecules to the surface and local substrate depletion [14].

Peptide-protein binding sites for YHNN and QYHH were determined by proteolytic mapping using reversible formaldehyde cross-linking [15]. YHNN and QYHH both bound to the same protein fragments (residues 419–447) at the subunit interface of β-Gal (Figure S6 in File S1). β-Gal from *E. coli* is only active in its tetrameric form [16], and it may be that YHNN and QYHH enhance the activity and stability of β-Gal by stabilizing its tetrameric structure.

**Figure 2:**
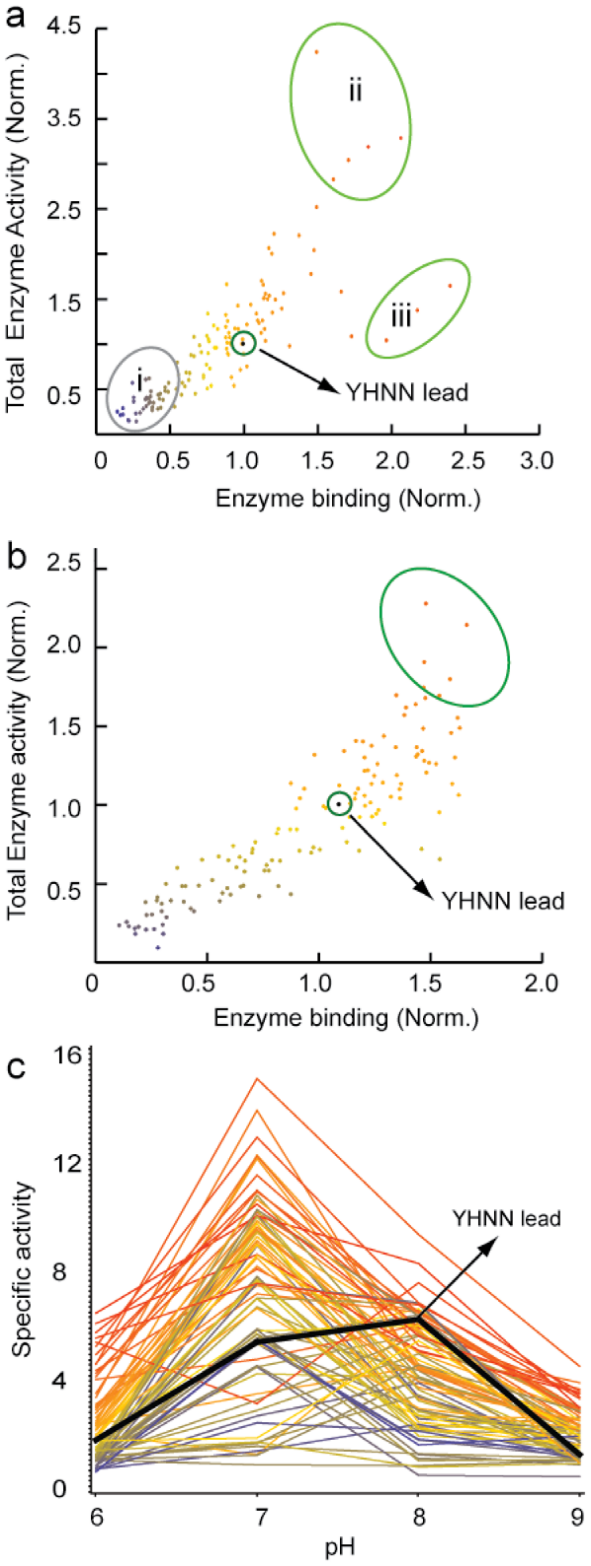
Point-variant screening of a lead peptide, YHNN. β-Gal was bound to a microarray containing 132 YHNN variants and its activity was measured. (a) The activity of bound β-Gal on microarrays as a function of the amount of enzyme bound to a particular variant feature at room temperature. (i) Variants with poor affinity and activity; (ii) Variants with stronger affinity and higher activity; (iii) Variants with stronger affinity but relative lower activity. All data is normalized to the binding and activity values for the lead peptide, YHNN. (b) Thermal-stability assay. β-Gal was bound to the microarray containing YHNN variants as in (a) at room temperature, followed by incubation in phosphate buffer at 55°C for one hour. Enzyme activity was then assayed at room temperature. The selection region (circled) contains variants that bind to the enzyme with higher relative specific activity (the ratio of binding to activity) under thermal stress compared to YHNN after incubation at high temperature. (c) pH activity range assay. YHNN variant microarrays were bound to β-Gal as in (a) and incubated at room temperature in buffers with pHs ranging from 6 to 9 for one hour and then assayed for activity at the pH of incubation. The black line is the specific activity of β-Gal bound to the lead peptide, YHNN.

Point-variant screening [17], [18] was applied to the YHNN peptide to improve both the affinity and activity of bound enzyme. 132 single-point variants, containing all substitutions of the amino acid set {Y, A, D, S, K, N, V, W} in each of the 17 randomized positions, were synthesized, printed on a microarray and analyzed for affinity and activity. Figure 2a shows the binding level vs. activity of β-Gal for each single-point variant, normalized to the YHNN- lead peptide. Several variants increased both binding level and activity, (region ii), including variant V9Y (YHNNPGFR**Y**MQQNKLHHGSC) which increased binding by 1.5-fold and specific activity by nearly 3-fold compared to the YHNN- lead peptide (Table S2 in File S1). V9Y conjugated to an aminated microwell increased both the binding and the specific activity of immobilized β-Gal by ∼2-fold compared to YHNN. This corresponds to a total bound enzymatic activity on the V9Y-modified surface that is ∼12-fold greater than the NHS surface and more than 5-fold greater than the amine surface (Figure S7 in File S1). Combining two advantageous point mutations into a single peptide (e.g.V9Y and N13Y, Supplemental Table S2 in File S1) resulted in an increase in the affinity of the peptide for binding to β-Gal but did not significantly enhance the specific activity of bound enzyme compared to single-point variants.

The library of single-point variants was also screened for enhanced thermal or pH stability of immobilized β-Gal. For thermal stability screening, enzyme was bound to microarrays containing the 132 single-point variants, at room temperature, and then the arrays were incubated at 55°C for one hour and assayed for activity at room temperature. A few point variants improved the resulting activity of bound β-Gal by nearly 50% compared to the YHNN- lead peptide (Figure 2a, circled region, and Table S3 in File S1). pH stability was screened by incubating enzyme-bound arrays in buffers ranging from pH 6 to pH 9 for one hour and then assaying activity at the pH used for incubation. In Figure 2c, some variants were found to significantly improve the specific activity of bound β-Gal at both low (pH 6) and high (pH 9) pH compared to the YHNN- lead peptide (e.g. Q12A, YHNNPGFRVMQ**A**NKLHHGSC shows a 4.1-fold activity increase at pH 6 and a 2.8-fold increase at pH 9, Table S4 in File S1).

## Discussion

We describe a rapid, systematic and general approach for modifying a surface in such a way that an enzyme can both bind tightly to the surface and maintain or even enhance its activity. Peptides can be rapidly selected from microarrays and covalently conjugated to surfaces for capturing target proteins. Peptide-modified surfaces improve both the specific activity and stability of bound β-Gal compared to free enzyme or to conventional enzyme surface immobilization approaches. In addition, the affinity and activity of one of the peptide-modified surfaces was further improved by single-point variant screening. Variants were found that not only improved activity under normal conditions, but enhanced thermal stability and increased enzyme activity at extreme pH. The surface modification is also specific for a particular enzyme, and thus the binding and activity enhancement can be patterned, opening the door for the development of multi-enzyme systems that are organized using a top-down patterning of surface modification combined with self-assembly of enzymatic systems on those surfaces. This approach appears to be applicable to the immobilization of a wide variety of enzymes on surfaces with optimized performance, and provides a potential mechanism for the patterned self-assembly of multiple enzymes on surfaces.

## Materials and Methods

### Chemicals

Resorufin β-D-galactopyranoside (RBG) and Alexa Fluor 647 were purchased from Invitrogen (Eugene, OR). β-galactosidase (β-Gal, E.coli), polyvinyl alcohol (PVA, M.W.: 124,000∼186,000), 4-nitrophenyl phosphate (PNPP), Phosphate Buffered Saline (PBS) and Tris Buffered Saline (TBS) were obtained from Sigma (St. Louis, MO). BS^3^ (Bis[sulfosuccinimidyl] suberate), alkaline phosphatase-conjugated strepavidin and iodoacetyl resin were purchased from Pierce (Rockford, IL). Sulfo succinimidyl-4-(*N*-maleimidomethyl) cyclohexane-1-carboxylate (Sulfo-SMCC) was purchased from bioWORLD (Dublin, OH). Aminated microwell plates were ordered from Corning. A 4 mg/mL stock solution of β-Gal was prepared in 10 mM potassium phosphate buffer with 0.1 mM MgCl_2_ at pH 7.4.

### Enzyme immobilization on modified microwells

Peptides were conjugated to aminated microwell surfaces through the specific reaction between C-terminal cysteines and the maleimide-activated surfaces, as shown in Figure 1a. 10 mM SMCC was prepared in 1× PBS buffer, pH 7.4. Next, 30 µL of SMCC was added into each aminated microwell and incubated for one hour at room temperature. The microwell plate was then briefly washed with pure water three times. Then, 30 µL of a 300 µM peptide solution, prepared in 1× PBS pH 7.4 plus 1 mM TCEP, was added to the appropriate SMCC-activated microwells. The reaction was incubated for 4 hours at room temperature, in the dark. After the conjugation reaction was complete, the microwells were washed for 5 minutes in 1× TBST, three times, followed by three washes in water. To immobilize the enzyme on peptide-modified surfaces, 30 µL of 25 nM biotin-labeled β-Gal was incubated in the peptide-modified microwells for two hours in 10 mM phosphate buffer, pH 7.3 with 100 µM MgCl_2_ and 0.05% Tween 20 (v/v%), at room temperature. The microwells were washed for 5 minutes in 1× TBST, three times, followed by three washes in phosphate buffer. At this point, the β-Gal-bound microwells were ready for testing. β-Gal was labeled with biotin using EZ-Link Sulfo-NHS-Biotinylation Kit purchased from Pierce (labeling ratio: ∼two biotin per enzyme molecule). Figures S8–10 in File S1 show the detailed optimization procedures for peptide-modified surfaces.

Covalent attachment of β-Gal to NHS (*N*-Hydroxysuccinimide)-activated surfaces was performed using BS^3^ homogeneous amine-reactive cross-linker, as recommended by the manufacturer. First, 30 µL of 2 mg/mL BS^3^ prepared in 1× PBS, pH 7.4 was incubated with the aminated microwells for half an hour. Then, the microwells were briefly washed with nanopure water, three times, to remove unreacted BS^3^ molecules. Finally, 30 µL of biotin-labeled β-Gal was incubated with the microwells for one hour, which were then washed three times in 1× TBST, followed by three washes in phosphate buffer.

The activity assay of surface-bound β-Gal was performed on a SpectraMax M5 96-well plate reader (Molecular Device, Sunnyvale, CA) by adding 100 µL of 100 µM RBG into the wells. The relative amount of surface-bound β-Gal was measured using an enzyme linked immunosorbant assay (ELISA). β-Gal was first labeled with biotin. Alkaline phosphatase-conjugated strepavidin (0.4 mg/ml) was diluted at 1∶1000 in 1× PBS, 0.05% (v/v) Tween 20. Next, 30 µL of streptavidin solution was added to the β-Gal-bound wells and incubated for one hour at room temperature. The streptavidin solution was then removed and the plate was washed three times with TBST buffer and three times with TBS buffer. Then, 200 µL of 1 mM PNPP was added to each well. The alkaline phosphatase activity was subsequently measured by reading the absorbance increase at 405 nm on the M5 plate reader. The β-Gal binding level was determined from the activity of alkaline phosphatase-conjugated strepavidin bound to the wells.

### Determining Michaelis constants of immobilized β-Gal

The determination of the enzyme kinetic constants (K_M_ and k_cat_) of immobilized β-Gal was performed on peptide-modified iodoacetyl polyacrylamide resin (UltraLink, Pierce, 50–80 µm diameter). To modify the bead surface with peptide, peptide solutions were incubated with iodoacetyl resin for one hour in 50 mM Tris buffer, 5 mM EDTA, pH 8.5. The unreacted iodacetyl groups were then capped with 50 mM L-cysteine. The amount of peptide immobilized on a bead surface was determined by comparing the peptide concentration of the unbound fraction (the remaining free peptide concentration after binding to the surface) to the starting concentration through absorbance changes at 280 nm. β-Gal was captured on the peptide-modified beads using the same protocol which immobilized the enzyme in the microwells, above. The amount of bead-immobilized β-Gal was measured by comparing the protein concentration of the unbound fraction to the starting protein concentration, determined at 280 nm. K_M_ and V_max_ (and thus k_cat_, using the total enzyme concentration) of β-Gal immobilized on peptide-modified beads were determined by fitting the activity vs. substrate concentration curves in the GraphPad program using the fitting equation of “Y = V_max_*X/(*K_m_*+X)”.

### Peptide mapping to β-Gal

The specific regions at which the peptides YHNN and QYHH bind to β-gal were determined by reversible formaldehyde cross-linking, as described previously.^8,12^ 200 µL of a 150 µM peptide solution was first conjugated to 100 µL of UltraLink iodoacetyl resin using the method described above. To promote cross-linking, the peptide-modified resin was incubated with 200 µL of 500 nM β-Gal for two hours. 200 µL of 1% formaldehyde (v/v), prepared in 1× PBS, was added to the enzyme-bound resin for 10 mins. Then, the formaldehyde solution was removed quickly by centrifugation. The resin was washed three times with 1 mM Glycine, pH 2.5 to remove enzyme that did not undergo cross-linking. Proteolytic digestion was performed by incubating the enzyme-bound resin with 34 nM Glu-c in ammonium bicarbonate buffer, pH 8.5, overnight at 37°C. Then, the resin was washed again with Glycine, pH 2.5 to remove Glu-c and any fragments that did not undergo cross-linking. The formaldehyde cross-linking was reversed by incubating the resin with 20 µL nanopure water at 70°C overnight. Following cross-link reversal, 100 µL of nanopure water was added to the resin to dissolve the free Glu-c-digested peptide fragments. The solution was spun to the bottom of the spin-column and then dried, by evaporation, in a vacuum centrifuge. The dried sample was re-dissolved with 10 µL of 1∶1 acetonitrile:H_2_O containing 0.1% trifluoroacetic acid and saturated alpha-cyano-4-hydroxycinammic acid matrix. The sample was spotted on a standard MALDI-MS (Matrix-assisted laser desorption/ionization mass spectrometry) target plate, and analyzed using a Bruker Microflex MALDI-MS.

### Microarray fabrication

Peptide microarrays containing 132, 20-mer single-point variants of the YHNN peptide were generated using our established, in-house printing method [19]. Each microarray was prepared by robotically spotting peptides, in triplicate, on a glass slide possessing an amino-silane surface coating. Synthesized peptides (70% purity) were purchased from Sigma. The last three carboxy-terminal positions of each peptide constituted a glycine-serine-cysteine (GSC) linker, used for conjugating the peptides to amino-silane surfaces through the C-terminal cysteine via a maleimide linker, Sulfo-SMCC (Pierce, Rockford, IL). A Telechem Nanoprint60 was used to spot approximately 500 pL of 1 mg/mL peptide prepared in 1× PBS for each feature on glass slides with 48 Telechem series SMP2 style 946 titanium pins.

### Enzyme assays on PVA-coated arrays

Enzyme assays on the microarrays were performed as described in the previous work [13]. Briefly, a peptide microarray was first prewashed with surface cleaning solvent (7.33% (v/v) acetonitrile, 37% isopropyl alcohol and 0.55% trifluoroacetic acid in water ) and then treated with capping buffer (3% (v/v) BSA, 0.02% (v/v) mercaptohexanol, 0.05% (v/v) Tween20 in 1×PBS) to block any active SMCC linker on the array surface. The array was incubated with a solution containing 10 nM Alexa™ 647- labeled β-Gal for two hours, allowing the enzyme to bind with peptides on the array surface. After washing off unbound enzymes, a fluorescent substrate analogue (FDG) was mixed with a 5% PVA solution and spin-coated onto the array surface for monitor the enzyme activity. The FDG molecules (substrate) in the PVA layer were converted to fluorescein (product), by the active enzymes bound to specific peptides on the array surface. The fluorescein molecules remained localized because of the PVA viscosity. Both the relative binding level of Alexa™ 647-labeled enzyme and the relative amount of fluorescein produced during the incubation period were determined by dual color scanning. Each array experiment was repeated at least three times under the same conditions for statistical analysis.

## Supporting Information

File S1 Additional figures and tables.
(PDF)

## References

[1] Sheldon Roger A (2007). Enzyme Immobilization: The Quest for Optimum Performance.. Advanced Synthesis & Catalysis.

[2] Laurent N, Haddoub R, Flitsch SL (2008). Enzyme catalysis on solid surfaces.. Trends in Biotechnology.

[3] Mateo C, Palomo JM, Fernandez-Lorente G, Guisan JM, Fernandez-Lafuente R (2007). Improvement of enzyme activity, stability and selectivity via immobilization techniques.. Enzyme and Microbial Technology.

[4] Cha T, Guo A, Zhu X-Y (2005). Enzymatic activity on a chip: The critical role of protein orientation.. PROTEOMICS.

[5] Clarizia L-JA, Sok D, Wei M, Mead J, Barry C (2009). Antibody orientation enhanced by selective polymer–protein noncovalent interactions.. Anal Bioanal Chem.

[6] Kim J, Jia H, Wang P (2006). Challenges in biocatalysis for enzyme-based biofuel cells.. Biotechnology Advances.

[7] Jung Y, Kang HJ, Lee JM, Jung SO, Yun WS (2008). Controlled antibody immobilization onto immunoanalytical platforms by synthetic peptide.. Analytical Biochemistry.

[8] Devlin J, Panganiban L, Devlin P (1990). Random peptide libraries: a source of specific protein binding molecules.. Science.

[9] Fodor S, Read J, Pirrung M, Stryer L, Lu A (1991). Light-directed, spatially addressable parallel chemical synthesis.. Science.

[10] Naffin JL, Han Y, Olivos HJ, Reddy MM, Sun T (2003). Immobilized Peptides as High-Affinity Capture Agents for Self-Associating Proteins..

[11] Williams BAR, Diehnelt CW, Belcher P, Greving M, Woodbury NW (2009). Creating Protein Affinity Reagents by Combining Peptide Ligands on Synthetic DNA Scaffolds.. Journal of the American Chemical Society.

[12] Naffin JL, Han Y, Olivos HJ, Reddy MM, Sun T (2003). Immobilized Peptides as High-Affinity Capture Agents for Self-Associating Proteins.. Chemistry&Biology.

[13] Fu J, Cai K, Johnston SA, Woodbury NW (2010). Exploring Peptide Space for Enzyme Modulators.. Journal of the American Chemical Society.

[14] Arrio-Dupont M (1988). An example of substrate channeling between co-immobilized enzymes Coupled activity of myosin ATPase and creatine kinase bound to frog heart myofilaments.. FEBS Letters.

[15] Sutherland BW, Toews J, Kast J (2008). Utility of formaldehyde cross-linking and mass spectrometry in the study of protein-protein interactions.. Journal of Mass Spectrometry.

[16] Jacobson RH, Zhang XJ, DuBose RF, Matthews BW (1994). Three-dimensional structure of [beta]-galactosidase from E. coli.. Nature.

[17] Wells JA (1990). Additivity of mutational effects in proteins.. Biochemistry.

[18] Greving MP, Belcher PE, Diehnelt CW, Gonzalez-Moa MJ, Emery J (2010). Thermodynamic Additivity of Sequence Variations: An Algorithm for Creating High Affinity Peptides Without Large Libraries or Structural Information.. PLoS ONE.

[19] Boltz KW, Gonzalez-Moa MJ, Stafford P, Johnston SA, Svarovsk SA (2009). Peptide microarrays for carbohydrate recognition.. Analyst.

