# Supplementary material for: Peptide-Modified Surfaces for Enzyme Immobilization
Source: PLoS One. 2011 Apr 8;6(4):e18692. doi: 10.1371/journal.pone.0018692 (PMC3073003; doi:10.1371/journal.pone.0018692)
Supplement: File S1 — Additional figures and tables. (PDF) [file pone.0018692.s001.pdf]

SUPPORT INFORMATION

Peptide-Modified Surfaces for Enzyme Immobilization

Jinglin Fu[1,2,3], Jeremy Reinhold[1,3] and Neal W. Woodbury[1,3]*

Center for Single Molecule Biophysics[1] and Center for Innovations in Medicine, the Biodesign Institute[2], Department of Chemistry and Biochemistry, Arizona State University, Tempe, AZ 85287[3]

E-mail: nwoodbury@asu.edu

*Address correspondence to: Neal W. Woodbury, 1001 S. McAllister Avenue, Tempe, AZ85287-5201. Phone: 480-965-6671, Fax: 480-727-0396

SUPPLEMENTARY TABLES

Table S1 Microarray data corresponding to the peptides selected for surface conjugation [a].

Peptide	Sequence	pI	Enzyme activity (Norm.)	Enzyme binding (Norm.)	Surface specific activity (Norm.)
1	YHNNPGFRVMQQNKLHHGSC	9.3	92.3	37.8	10.9
2	QYHHFMNLKRQGRAQAYGSC	9.8	90.4	42.4	9.5
3	RVFKRYKRWLHVSRYYFGSC	10.3	0.9	50.3	0.08
4	PASMFSYFKKQGYYYKLGSC	9.4	2.3	63.9	0.16
5	EFSNPTAQVFPDFWMSDGSC	3.5	0.7	0.4	-

[a] Peptides 1 and 2 were selected to promote strong activity when bound to β-Gal. Peptides 3 and 4 bind to β-Gal but result in very low enzyme activity. Peptide 5 does not bind to β-Gal. Surface specific activity of β-Gal bound to each of the peptide spots was calculated by dividing the total bound enzyme activity by the total binding intensity. The detailed selection is described in reference [13].

Table S2 Selected point variants that improve the binding affinity and activity of β-Gal on a microarray surface [a].

	Sequence	Binding	Total activity	Surface Specfic activity
1	YHNNPGFRVMQQNKLHHGSC	1.00	1.00	1.00
2	YHNSPGFRVMQQNKLHHGSC	1.22 ± 0.15	1.89 ± 0.19	1.55
3	YHNNPGYRVMQQNKLHHGSC	1.77 ± 0.14	3.15 ± 0.29	1.78
4	YHNNPGFYVMQQNKLHHGSC	0.93 ± 0.20	1.55 ± 0.22	1.67
5	YHNNPGFRYMQQNKLHHGSC	1.50 ± 0.12	4.25 ± 0.18	2.82
6	YHNNPGFRWMQQNKLHHGSC	1.62 ± 0.13	2.83 ± 0.1	1.75
7	YHNNPGFRVYQQNKLHHGSC	1.72 ± 0.15	3.04 ± 0.26	1.77
8	YHNNPGFRVMQQYKLHHGSC	1.50 ± 0.21	2.52 ± 0.40	1.68
9	YHNNPGFRVMQQNKWHHGSC	2.10 ± 0.15	3.29 ± 0.20	1.57
10	YHNSPGFRYMQQNKLHHGSC	1.62 ± 0.16	3.53 ± 0.26	2.18
11	YHNNPGYRYMQQNKLHHGSC	2.35 ± 0.22	6.01 ± 0.40	2.56
12	YHNNPGFYYMQQNKLHHGSC	0.66 ± 0.11	0.91 ± 0.10	1.37
13	YHNNPGFRYMQQYKLHHGSC	2.20 ± 0.20	4.78 ± 0.50	2.17
14	YHNNPGFRYMQQNKWHHGSC	2.05 ± 0.26	5.72 ± 0.61	2.79

[a] Peptide 1 is the YHNN lead; Peptides 2-9 are selected single-point variants of the YHNN lead; Peptides 10-14 are combinations of two single-point variants. The combinations of two selected single-point variants sometimes improve the affinity to β-Gal, but do little to enhance the specific activity of the bound enzyme. All binding and activity data is normalized to that of the YHNN lead.

Table S3 Point variants that improve the thermal stability of bound β-Gal [a].

Sequence	Binding (Norm.) at 55 C	Specfic activity (Norm.) at 55 C
WHNNPGFRVMQQNKLHHGSC	1.28	1.45
YHSNPGFRVMQQNKLHHGSC	1.48	1.42
YHNNPGYRVMQQNKLHHGSC	1.77	1.38
YHNNPGWRVMQQNKLHHGSC	1.07	1.35
YHNNPGFRYMQQNKLHHGSC	1.5	1.86
YHNNPGFRVMQQNKLAHGSC	0.89	1.54
YHNNPGFRVMQQVKLHHGSC	1.67	1.47

[a] All data is normalized to the YHNN lead.

Table S4 Point variants that modulate the optimal pH range of bound β-Gal [a].

Sequence	Specific activity at pH 6	Specific activity at pH 7	Specific activity at pH 8	Specific activity at pH 9
YHANPGFRVMQQNKLHHGSC	2.53	1.37	1.05	2.71
YHNSPGFRVMQQNKLHHGSC	3.08	1.58	0.95	3.30
YHNNPGYRVMQQNKLHHGSC	2.68	1.58	0.80	3.46
YHNNPGWRVMQQNKLHHGSC	2.13	1.82	0.84	3.70
YHNNPGFRYMQQNKLHHGSC	3.47	1.99	1.09	3.39
YHNNPGFRSMQQNKLHHGSC	3.61	1.47	0.90	3.29
YHNNPGFRVMAQNKLHHGSC	3.83	1.33	0.81	3.31
YHNNPGFRVMNQNKLHHGSC	2.04	1.34	0.69	1.89
YHNNPGFRVMQANKLHHGSC	4.08	1.91	1.35	2.76
YHNNPGFRVMQNNKLHHGSC	3.27	1.42	1.11	3.11
YHNNPGFRVMQQAKLHHGSC	2.85	1.53	1.02	2.55
YHNNPGFRVMQQNKNHHGSC	2.85	1.49	1.53	4.33

[a] All data is normalized to the YHNN lead.

SUPPLEMENTARY FIGURES

Figure S1

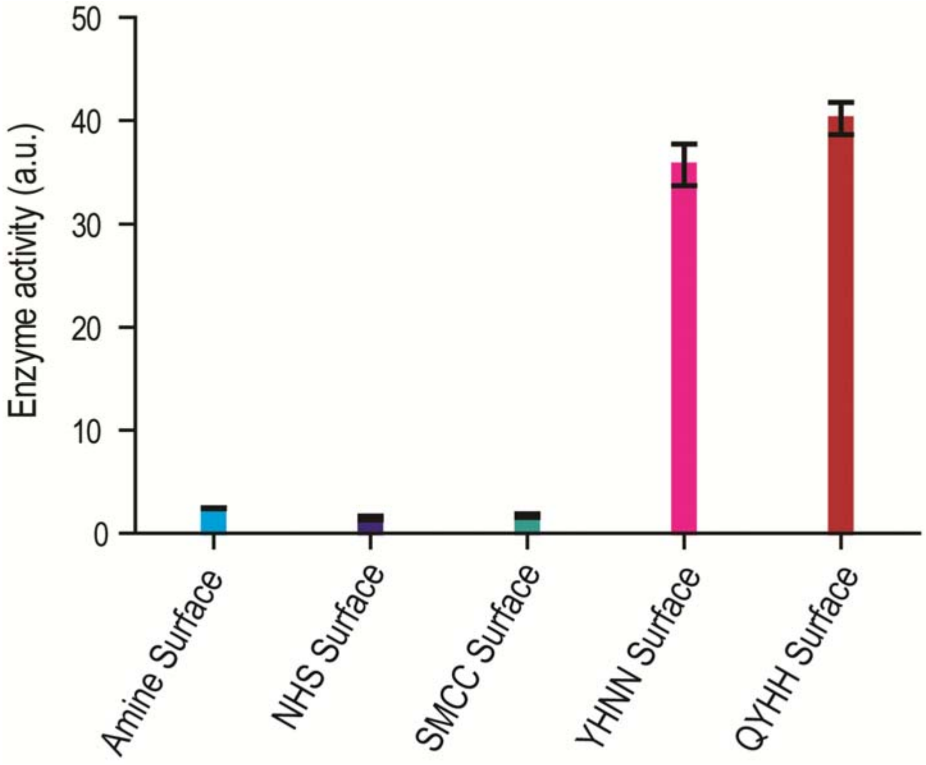

Figure S1 Specificity of different types of surfaces for capturing β-Gal in a protein mixture. 25 nM β-Gal was mixed with 3 % BSA and then applied to these surfaces. The activity of each enzyme-bound surface was measured by adding 100 µL of 100 µM RBG into each microwell.

Figure S2

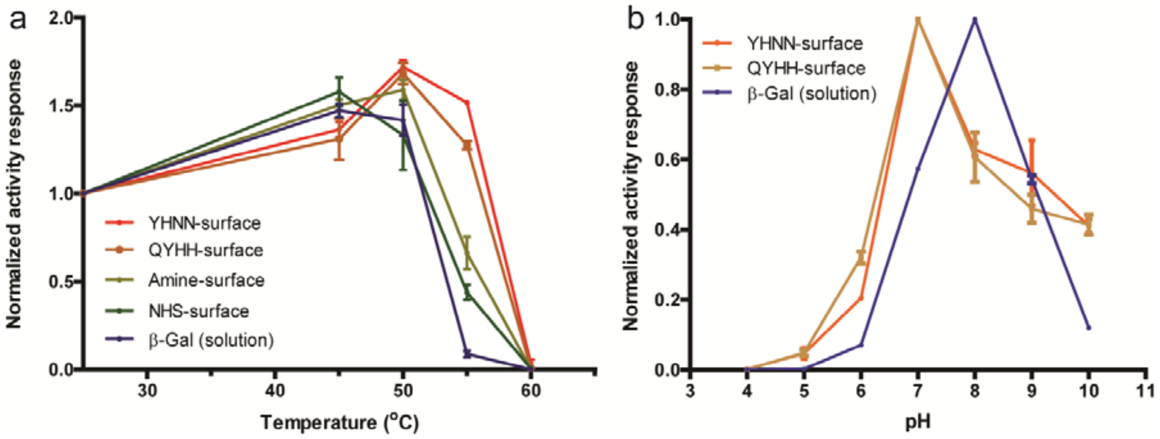

Figure S2. (a) Normalized thermal stability of immobilized β-Gal on different surfaces. The thermal stability of immobilized β-Gal was measured by incubating the enzyme at a specific temperature between 25 °C and 60 °C for one hour and then assaying its activity. Note that the activity of β –Gal after exposure to high temperature is substantially enhanced when bound to the YHNN and QYHH peptide-modified surfaces. The activities of β-Gal at different temperatures were normalized to that at 25°C. (b) Normalized activity of β-Gal immobilized on peptide-modified surfaces as a function of pH. The solution pH dependence of the activity is shown in dark blue for comparison. The activities of β-Gal at different pHs were normalized to the maximum activity at optimal pH.

Figure S3

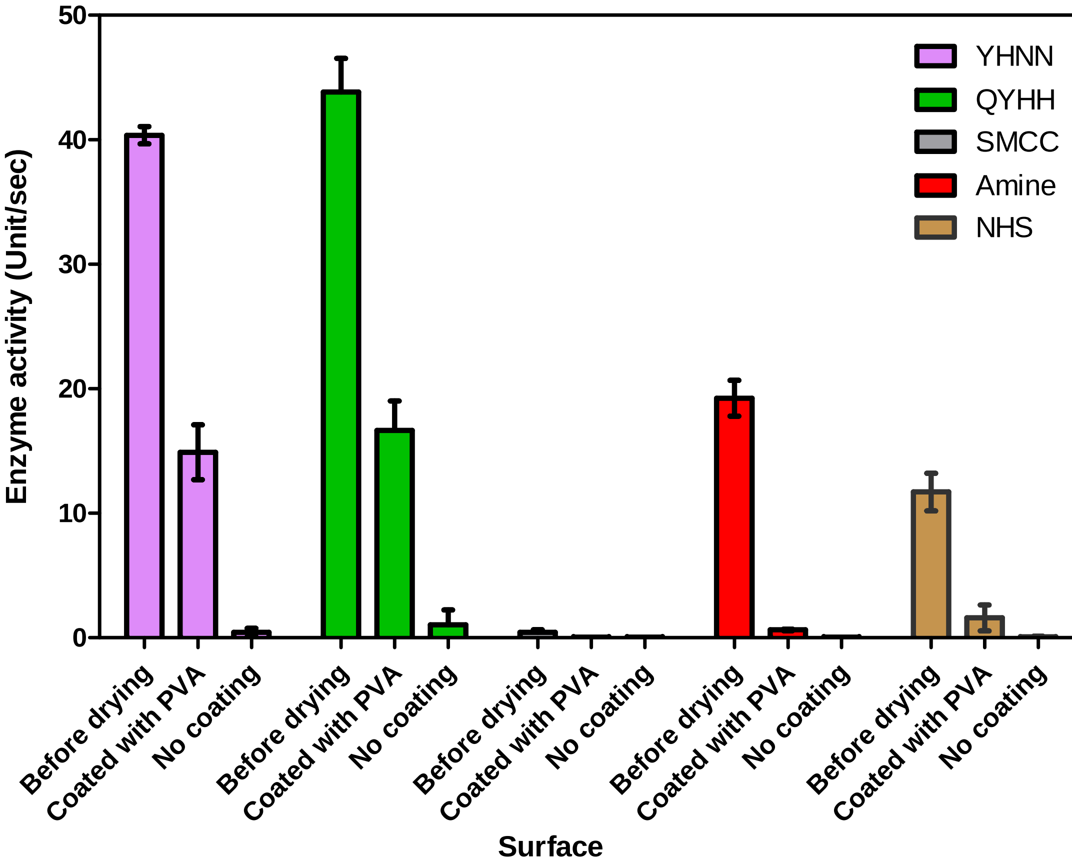

Figure S3 Long-term stability of β-Gal immobilized on different surfaces. The long-term stability of the enzyme was tested in two ways. One method used was to coat the enzyme-bound microwell with PVA, dry it and then store it at room temperature in this condition for a week (the second column for each sample). The other method used was to dry the enzyme-bound microwell without PVA coating and store it at room temperature for a week (the third column for each sample). The remaining enzyme activity after storage is assayed by adding 100 μL of 100 μM RBG into each well. As shown in the figure, the peptide surfaces combined with PVA coating greatly maintain the enzyme activity after storage compared to the other surfaces.

Figure S4

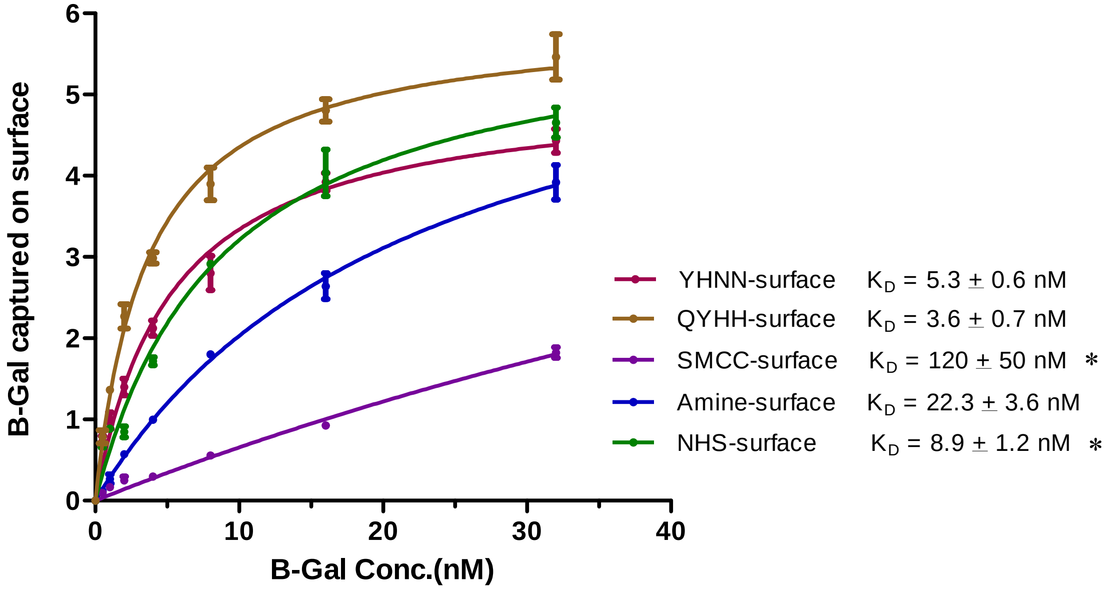

Figure S4. The apparent dissociation constants of different surfaces determined by ELISA. All data is fitted using the program GraphPad and the fitting equation: $Y=B_{max}*X/(Kd + X)$, where B_{max} is the theoretical maximum binding level of β-Gal. [*] For the SMCC- and NHS-surfaces, there is no real K_D since this is a covalent conjugation with no dissociation equilibrium. In that case, the apparent K_D is calculated from curve fitting and likely reflects the saturation of enzyme attachment to the NHS-surface.

Figure S5

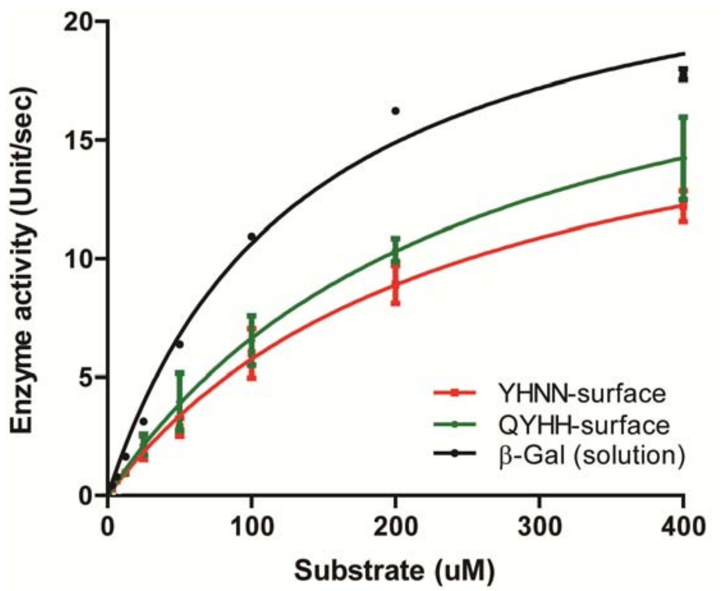

Figure S5. Colored curves: Curve fit determining the Michaelis constants of β-Gal immobilized on YHNN- and QYHH-modified beads using an amount of bound enzyme equivalent to a solution enzyme concentration of ~ 150 pM. Black curve: Michaelis constans determined for the free enzyme at 150 pM. The enzyme activities are measured as a function of the concentration of the substrate RBG, between 3 μM and 400 μM, at 25 ^{o}C. All data is fitted using the program GraphPad and the fitting equation: $Y=V_{max}*X/(K_m+X)$

Figure S6

a

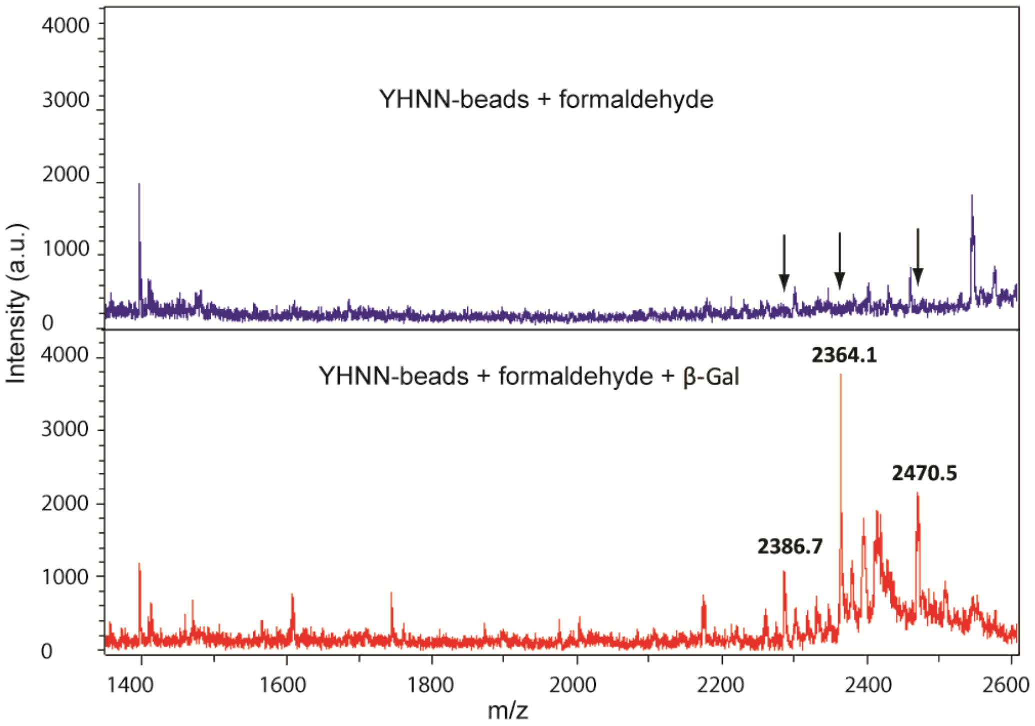

Observed Mass (Da)	β-Gal Fragment	Fragment Sequence	Oxidation
2260.2	430-447	PRWLPAMSERVTRMVQRD	2
2286.7	470-487	ALYRWIKSVDPSRPVQYE	5
2363.9	419-438	GMVPMNRLTDDPRWLPAMSE	3
2379.8	419-438	GMVPMNRLTDDPRWLPAMSE	4
2395.9	419-438	GMVPMNRLTDDPRWLPAMSE	5
2411.9	419-438	GMVPMNRLTDDPRWLPAMSE	6
2422.2	429-447	DPRWLPAMSERVTRMVQRD	5
2370.5	429-447	DPRWLPAMSERVTRMVQRD	8

b

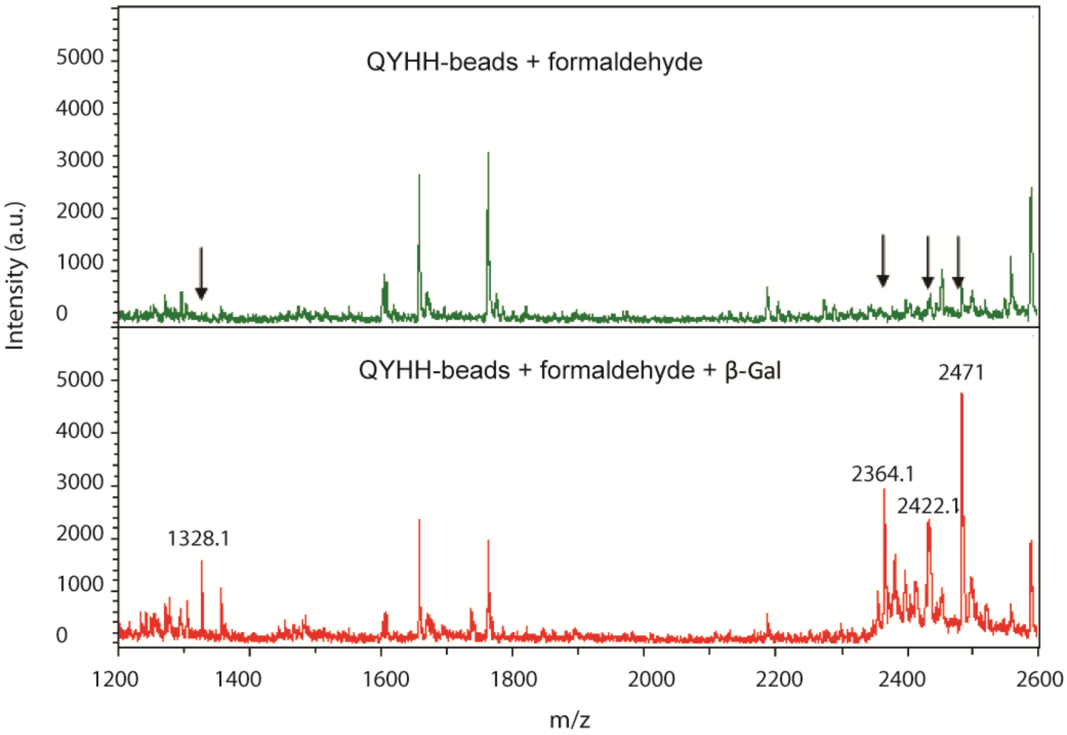

Observed Mass (Da)	β-Gal Fragment	Fragment Sequence	Oxidation
1328.2	419-429	GMVPMNRLTDD	5
2355.6	430-447	PRWLPAMSERVTRMVQRD	8
2364.1	419-438	GMVPMNRLTDDPRWLPAMSE	3
2380.4	419-438	GMVPMNRLTDDPRWLPAMSE	4
2396.5	419-438	GMVPMNRLTDDPRWLPAMSE	5
2412.6	419-438	GMVPMNRLTDDPRWLPAMSE	6
2422.1	429-447	DPRWLPAMSERVTRMVQRD	5
2471	429-447	DPRWLPAMSERVTRMVQRD	8

Figure S6 MALDI-TOF analysis of β-Gal crosslinked to peptide-modified beads after Glu-c digestion. (a) The upper figure is a YHNN-bead blank control and the lower figure is β-Gal crosslinked to YHNN-beads. The protein fragment analysis indicates that the main binding region between the enzyme and the YHNN-beads is located near the amino acid residues 419-447 (bottom table). Many of the digest fragments observed are actually from the same stretch of amino acid sequence but have different different degrees of oxidation as a result of exposure to formaldehyde. (b) The upper figure is a QYHH-bead blank control and the lower figure is β-Gal crosslinked to QYHH-beads. The mapping data shows that the binding region of the peptide QYHH with β-Gal is near amino acid residues 419-447, just as was observed for the peptide YHNN. This region of β-Gal is located near the interface between two subunits of the protein.

Figure S7

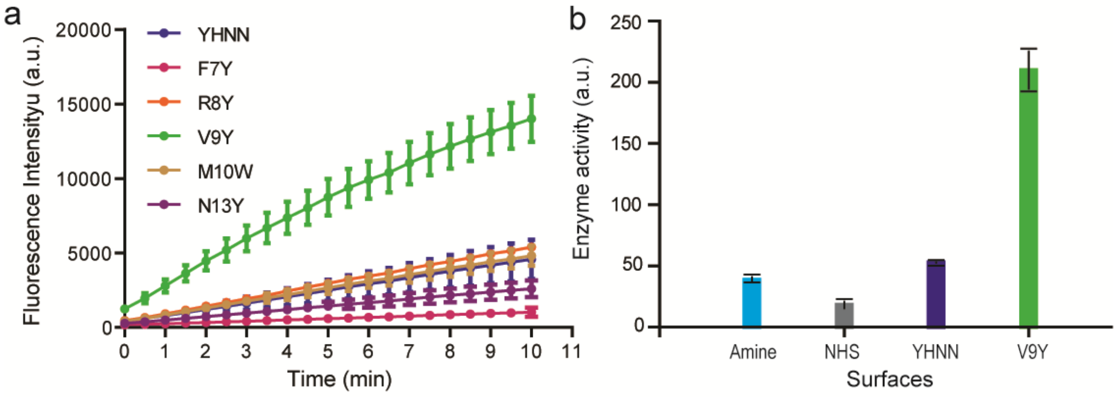

c

Sequence		β-Gal Binding	Total β-Gal Activity	Surface Specific Activity
YHNN	YHNNPGFRVMQQNKLHHGSC	2.0 ± 0.3	6.2 ± 2.1	3.1
F7Y	YHNNPGYRVMQQNKLHHGSC	1.1 ± 0.2	1.5 ± 0.5	1.3
R8Y	YHNNPGFYVMQQNKLHHGSC	2.0 ± 0.3	8.8 ± 0.5	4.4
**V9Y**	YHNNPGFRYMQQNKLHHGSC	4.2 ± 0.2	27.2 ± 3.6	6.5
M10W	YHNNPGFRVWQQNKLHHGSC	1.9 ± 0.2	3.9 ± 0.9	2.1
N13Y	YHNNPGFRVMQQYKLHHGSC	1.9 ± 0.2	7.4± 0.8	3.9

Figure S7 Activity of β-Gal immobilized on different surfaces including ones in which the lead peptide, YHNN, was modified by point variants selected from microarray screening. (a) YHNN and 5 selected single-point variants were used to modify aminated microwell surfaces for capturing β-Gal. 3 variants, R8Y, V9Y and N13Y showed improvement of total bound enzyme activity. The variant V9Y demonstrated the best enzyme immobilization performance: a roughly 2-fold improvement both in surface affinity and relative specific activity of the bound enzyme, and nearly a 5-fold improvement in total activity of the bound enzyme. (b) Total enzyme activity immobilized on the YHNN- and V9Y-modified surfaces compared to amine and NHS surfaces. (c) Binding and activity of β-Gal immobilized on surfaces modified by peptides. The relative β-Gal binding amount is determined by measuring the activity of APase conjugated streptavidin bound to the biotinlyted β-Gal. The enzyme activity is evaluated by adding 100 µL of 100 µM RBG into each microwell.

Figure S8

a

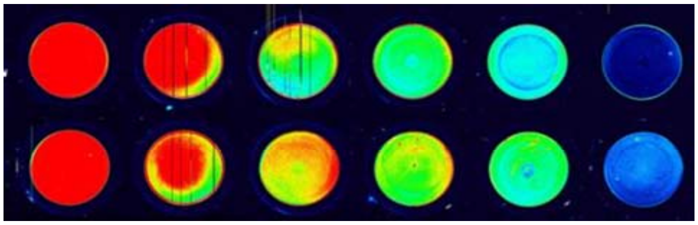

b

Sample	Raw intensity (Alexa647)	Conjugation Yield
Amine surface	30757	0.00
0.675 mM SMCC	19035	0.38
1.25 mM SMCC	16116	0.48
2.5 mM SMCC	12008	0.61
5 mM SMCC	7813	0.75
10 mM SMCC	3025	0.90
20 mM SMCC	1356	0.96

c

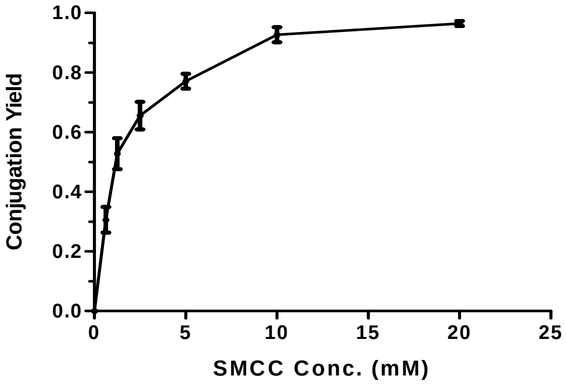

Figure S8. Optimization of SMCC conjugation: (a) raw fluorescence images of Alexa-647-labeled aminated microwells with SMCC capping at different concentrations. SMCC will conjugate to the amine groups on the microwells and prevent the labeling of Alexa-647 dye molecules. The higher the conjugation yield, the less the fluorescence from the labeled fluorophores; (b) Fluorescence intensities of Alexa-647-labeled microwells with different concentrations of SMCC capping; (c) surface conjugation yields of SMCC at different concentrations. The result shows that a 10 mM SMCC solution can be used to achieve a surface conjugation yield of more than 90%.

Figure S9

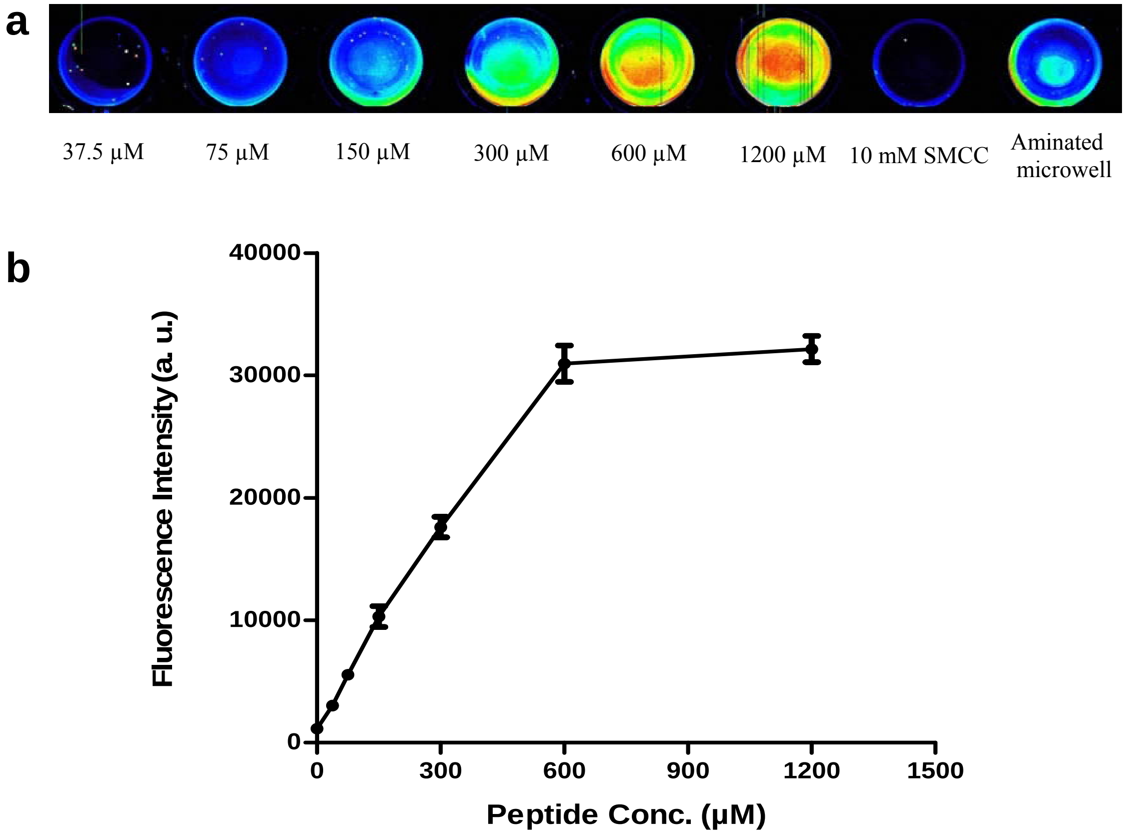

Figure S9. Optimization of peptide concentration for surface conjugation: (a) raw fluorescence images of Alexa-647-labeled microwells conjugated with different concentrations of peptide solution. 10 mM SMCC was first used to activate the aminated microwells, and then peptides were conjugated to SMCC-activated surfaces through specific reactions between the C-terminal cysteine and maleimide. Surface peptide densities were measured by labeling the microwells with amine-reactive Alexa 647. The more peptide on surface, the stronger the labeled fluorescence intensity due to the reaction between the dye and the peptide amine groups. (b) Surface fluorescence intensity as a function of peptide concentration used for conjugation. The peptide used is YHNN with a sequence of "YHNNPGFRVMQQNKLHHGSC".

Figure S10

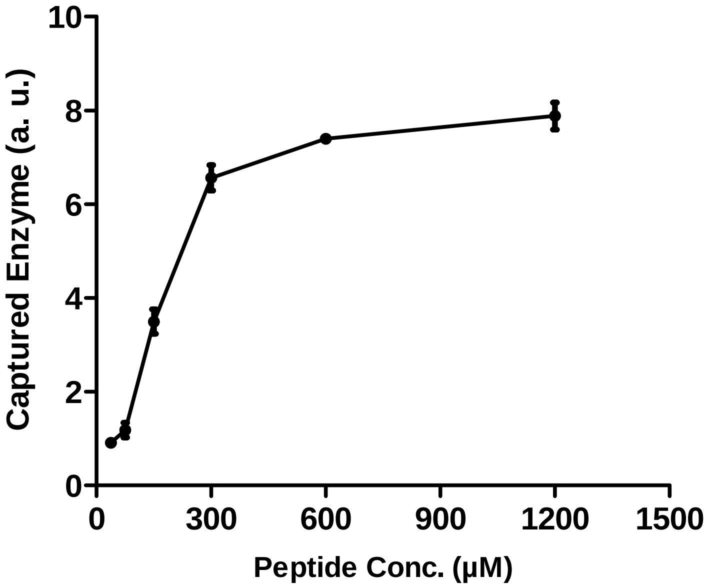

Figure S10. Optimization of peptide density for capturing β-Gal on peptide-modified microwells. Different densities of surface-immobilized peptide were created by varying the concentrations of peptide solution used for immobilization. The amount of immobilized β-Gal started to get saturation when surface was modified with 300 μM or higher concentrations of peptide solution. The peptide used for optimization was YHNN.
